# The role of Food Science and Foodomics in the implementation of One-Health

**DOI:** 10.3389/fnut.2025.1662767

**Published:** 2025-09-17

**Authors:** Elena Ibáñez, Zhaowei Zhang, Alejandro Cifuentes

**Affiliations:** ^1^Foodomics Lab, CIAL, CSIC, Madrid, Spain; ^2^School of Bioengineering and Health, Wuhan Textile University, Wuhan, China

**Keywords:** One-Health, Foodomics, Food Science, SDGs, synthetic biology

## Abstract

The concept of One-Health recognizes that the health of people is closely connected to the health of animals and our shared environment. Despite the most frequent applications of One-Health approaches have been in the control of parasitic and infectious (zoonotic) diseases shared between humans and animals, many other areas of implementation should be explored that will help to overcome some of the current planetary challenges. In this work, we discuss for the first time the multiple possibilities of advancing the development of One-Health through future applications of Food Science and Foodomics, showing a vast and unexplored area of research. We also discuss the emerging potential of synthetic biology for Food Science and how it can help to achieve the One-Health goals.

## 1 Introduction

### 1.1 One-Health concept

The world is facing important challenges related with climate change, sustainable food systems and the roadmap for achieving the Sustainable Development Goals (SDGs) by 2030. Many of these goals can be linked through the new concept of One-Health (18). “One-Health” is an integrated, unifying and transdisciplinary approach to sustainably balance and optimize the health of people, animals and ecosystems. It recognizes the health of humans, domestic and wild animals, plants, and the wider environment (including ecosystems) are closely linked and inter-dependent. This term was first used in 2003–2004 in response to the emergence and spread of two highly pathogenic viruses: SARS (severe acute respiratory syndrome) and H5N1 (avian influenza). These events clearly demonstrated that human health is inseparable from the health of animals and the broader environment. The approach mobilizes multiple sectors, disciplines and communities at varying levels of society to work together to foster wellbeing and tackle threats to health and ecosystems, while addressing the collective need for clean water, energy and air, safe and nutritious food, taking action on climate change, and contributing to sustainable development.

### 1.2 Current challenges: SDGs, food, and One-Health

The United Nations SDGs (1) are strongly linked to the concept of One-Health as they include targets for health and wellbeing, clean water and sanitation, climate action, as well as sustainability in marine and terrestrial ecosystems. The One-Health concept transcends anthropocentrism, attempting to simultaneously provide optimal health for humans, animals, and the environment, following a sustainable development (2).

In this regard, the One-Health High-Level Expert Panel (OHHLEP) (3), [i.e., the scientific and strategic advisory group to the Quadripartite organizations—the Food and Agriculture Organization of the United Nations (FAO), the United Nations Environment Program (UNEP), the World Health Organization (WHO) and the World Organization for Animal Health (WOAH)] has defined five key underlying principles, namely:

1. Equity between sectors and disciplines;
2. Socio-political and multicultural parity (the doctrine that all people are equal and deserve equal rights and opportunities) and inclusion and engagement of communities and marginalized voices;
3. Socioecological equilibrium that seeks a harmonious balance between human–animal–environment interaction and acknowledging the importance of biodiversity, access to sufficient natural space and resources, and the intrinsic value of all living things within the ecosystem;
4. Stewardship and the responsibility of humans to change behavior and adopt sustainable solutions that recognize the importance of animal welfare and the integrity of the whole ecosystem, thus securing the wellbeing of current and future generations;
5. Transdisciplinary and multi-sectorial collaboration, which includes all relevant disciplines, both modern and traditional forms of knowledge and a broad representative array of perspectives.

The effects of human activity on our environment and planetary boundaries have a profound impact on the health and wellbeing of humans, animals and the ecosystems we co-habit. The OHHLEP has identified a series of societal, animal and environmental challenges stemming from inter-linked categories of human activity (anthropogenic influences on health) (4). The listed challenges are general in most instances and described as factors contributing to risks and vulnerability to poor health for humans, animals and ecosystems. Their relevance and scale of influence varies based on the specific context. They are intended to convey key themes, to be more precisely grouped and standardized in future iterations as the evidence base matures (4). Interestingly, among the 60 challenges described (4), few are directly related to Food Science or food systems, namely:

- Inequitable access to safe and nutritious food
- Increasingly complex products/food system trade
- Inappropriate use of antimicrobials, pesticides and insecticides
- Unsustainable agricultural intensification
- Intensified aquaculture, livestock and wildlife farming systems
- Unsustainable growth in livestock/poultry populations and density

Besides, several of these 60 challenges are also indirectly related to Food Science and food systems since they may have a clear impact on food or, *vice versa,* the food systems may have a clear impact on these challenges, namely:

- Poor biosecurity
- Genetic diversity and breed loss
- Poor conditions and standards of animal welfare and protection
- Selective breeding of genetic traits that compromise animal health and welfare
- Wide disparities in access to effective medical technologies for animal health
- Land use change
- Biodiversity loss
- Unsustainable harvest of wild species
- Growing landfill/non-recyclable waste
- Primary and secondary forest loss and unrestrained monoculture expansion

Despite the most frequent application of the One-Health approach being in the control of parasitic and infectious (zoonotic) diseases shared between humans and animals, it has also been mentioned that there is a lack of integration between the One-Health and Food System approaches to manage zoonoses (5). Moreover, many other areas of implementation should be explored, ideally following a deeper procedure.

## 2 Discussion: implementing One-Health through Food Science and Foodomics

**Figure 1:**
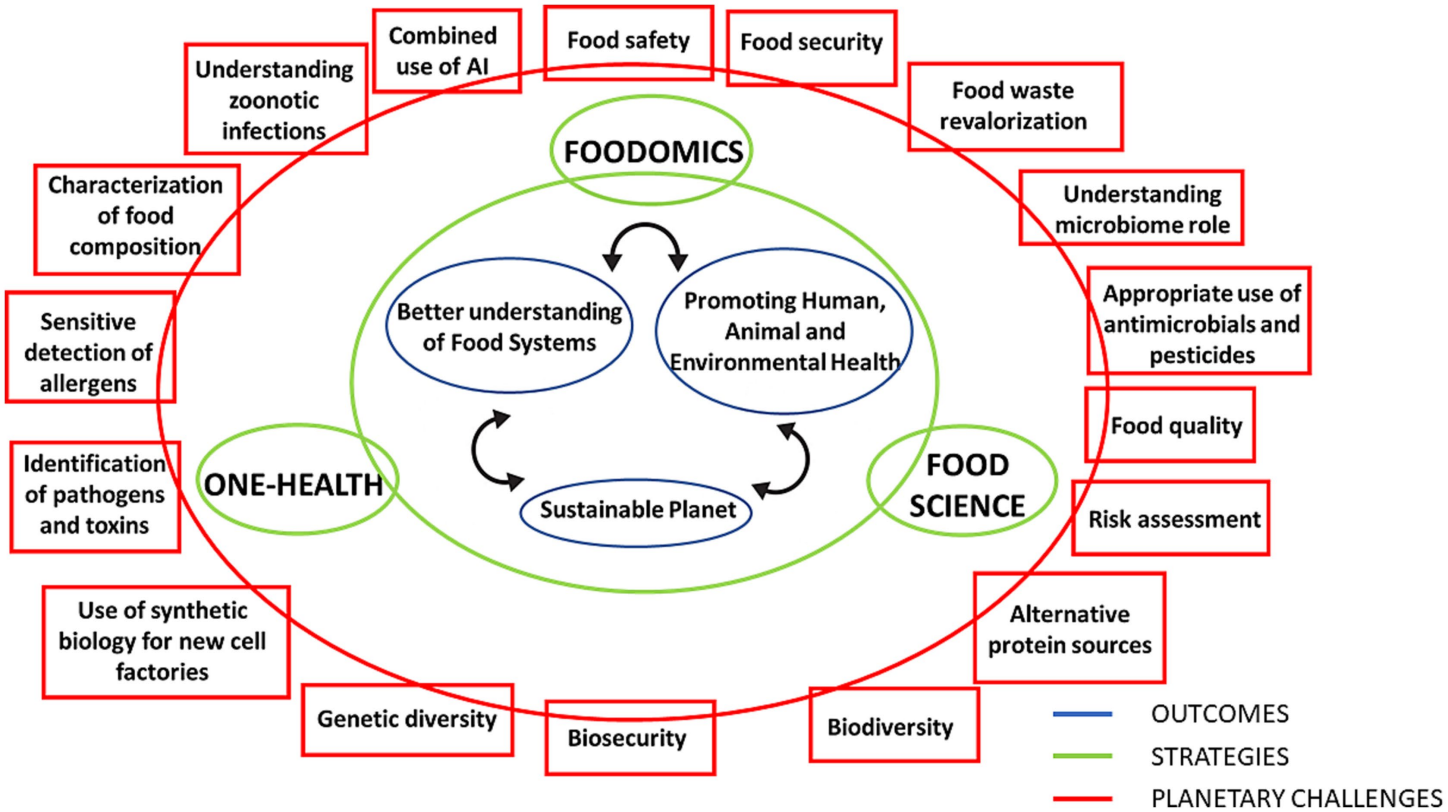
Planetary challenges, strategy proposed in this work combining One Health-Food Science-Foodomics, and expected outcomes.

Interestingly, the contribution of the binomial “food bioactive & human health” has been scarcely discussed under the frame of the One-Health principles, as can be deduced from the number of papers published following this idea [zero, according to a search done in the ISI Web of Knowledge in January 2025 using “one health” AND (“food bioactivity” OR “food bioactive”)]. This is due to the One-Health concept, although formulated two decades ago, remains challenging to implement. In the following, we will discuss the possibilities of advancing One-Health implementation through future applications of Food Science and Foodomics, showing the potential of this unexplored area of research that may help to solve many of the planetary challenges indicated in Figure 1. Moreover, this combination (Food Science-Foodomics-One-Health) can provide important outcomes in terms of food systems understanding, planet sustainability and health of humans, animals and environment (see Figure 1).

In a recent opinion article from the Editorial Team of *Exploration of Foods and Foodomics* journal (6), an interesting comparison about the perspective of One-Health from Europe, China and Latin America was presented. One of the key aspects rely on how the loss of biodiversity affects directly human health, and indirectly through changes in food availability and nutritional quality, but also contributes to increase zoonotic diseases while disrupting healthy ecosystems (including clean air and water, regulation of climate and infection diseases). In terms of human health, chronic diseases (such as cardiovascular diseases and cancer) are considered the major public health problems in Western countries, while China is more focused on obesity and malnutrition linked to agriculture resilience and productivity lost. From Latin America, the One-Health perspective is more holistic and has important differences compared to Europe and China; among them, regions’ rich biodiversity, traditional knowledge about sustainable food practices and medicinal plants, and the need of preservation of cultural heritage. By prioritizing sustainable land and water management, Latin America can create a more resilient food system that supports both biodiversity and the health of its populations (encountering Western diseases together with infectious diseases and malnutrition). This holistic approach is essential for tackling the intertwined challenges of food security, environmental degradation, and public health.

### 2.1 A roadmap for advancing One-Health via Food Science

**Figure 2:**
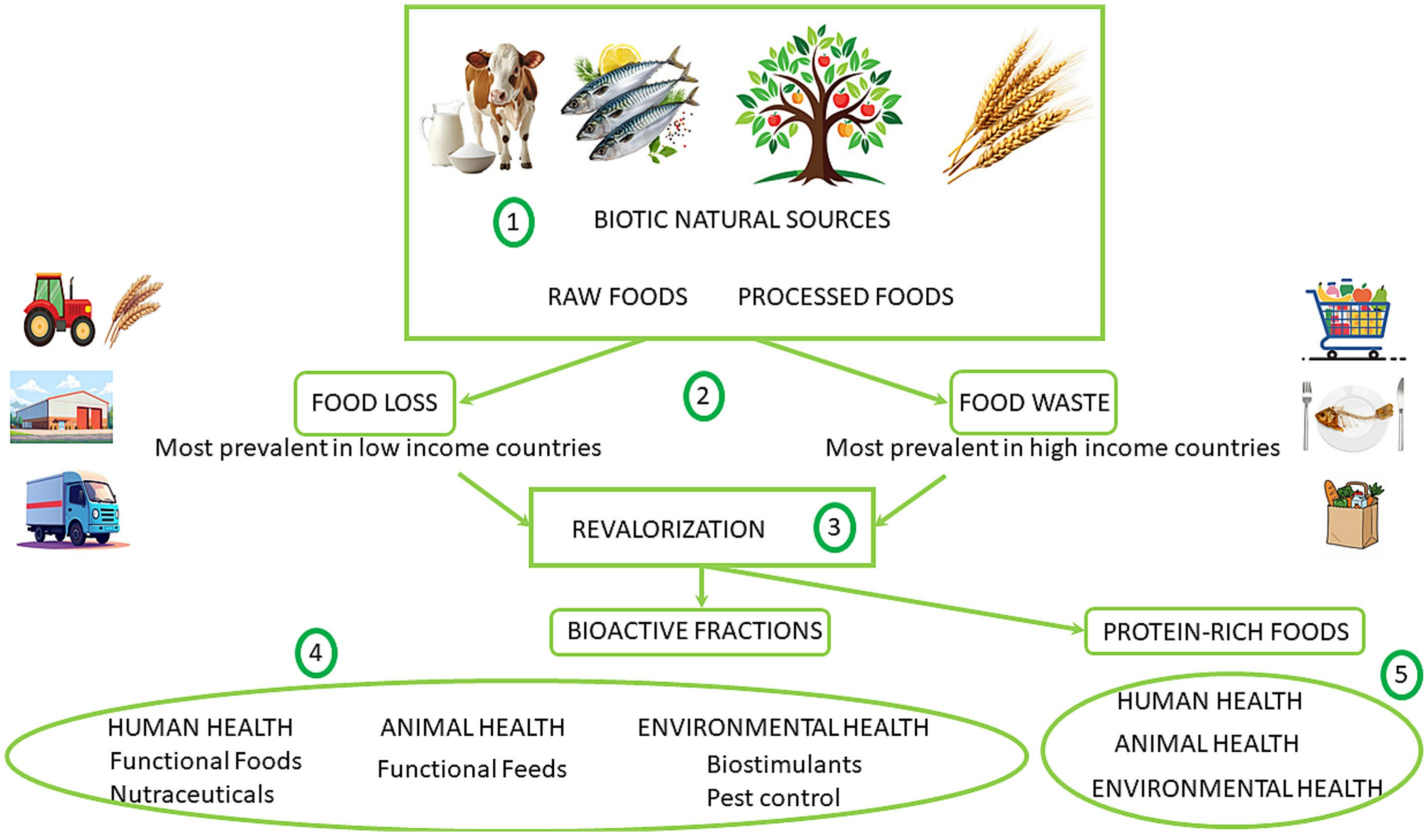
Example of a proposed roadmap for advancing the implementation of One-Health via Food Science.

Among the multiple possibilities for advancing One-Health implementation via Food Science, the following roadmap is proposed (see Figure 2): 1) biotic natural sources (vegetal, animal) are employed as foods either raw (after farming, breeding, harvesting or fishing) or after processing; 2) food loss and food waste both represent a reduction in the quantity and/or quality of edible food, but they occur at different stages of the food supply chain. Food loss typically happens during production, harvest, postharvest, storage and transportation. Food waste occurs during distribution and consumption, either at retail, food service, or household levels, and often involves discarding perfectly edible food. Food loss is most prevalent in lower income countries when food is, for example, unintentionally damaged or destroyed by pests or mold. Food waste results from decisions and actions by retailers, food service providers and consumers; it happens most often in high-income countries at restaurants, hotels and homes; 3) valorizing these food loss and food wastes can improve the sustainability of our food systems, including the resources needed to produce the food (water, land, energy, labor, capital); moreover, it can help improving environmental health since disposal of food loss and waste in landfills lead, among others, to greenhouse emissions, contributing to climate change; 4) one way of revalorizing is through the production of bioactive fractions with beneficial effects on human health (elaborating functional foods and/or nutraceuticals with therapeutic potential), animal health (for producing functional feed with antiparasitic activities or to improve health status), environmental health (for biostimulant, pest control, etc.); 5) another way of revalorizing these wastes or wastes’ residues is through its use (after proper processing) as protein-rich foods that can also provide benefits for human health (a better and more straightforward access to proteins), animal health (for animals’ growing, for instance for insects’ cultivation) and environmental health (including the reduction on agriculture intensification for cattle/poultry raising and the associated decrease on greenhouse emissions).

Comi et al. (7) discuss the link between the SDGs and One-Health concept as starting point for selecting the most interesting agro-food waste as supply chain for producing nutraceuticals. Important aspects here to be considered are: the supply chain should be committed to sustainable practices, with an appropriate bioactive content with potential activity against the target disease, making use of possible innovative and sustainable technologies to obtain the active ingredients, the fulfillment of the Agenda 2030 and One-Health principles, the geographical proximity and certificates of commitment to environmental stewardship, quality control and social responsibility.

To illustrate the Figure 2 concept linking Food Science and One-Health, one supply chain fulfilling the above-mentioned requirements has been selected: apple waste and, more specifically, apple pomace (a waste by-product of the apple processing industry). Watanabe et al. (8), examined the effects of an extract from apple pomace on N-methyl-D-aspartate receptor antagonist MK-801-induced memory impairment in mice, showing that a repeated treatment with apple pomace extract for 7 days reversed the MK-801-induced impairment of associative memory and recognition memory, through alteration of the gene expression profile in the hippocampus of mice. Results showed that the extract from apple pomace induced upregulation of the mRNA expression for Zfp125 and Gstp1 and that gene sets related to synapse and neurotransmission were upregulated by the extract. Animal health has also been improved using apple pomace; in a recent study by Pistol et al. (9), apple pomace was employed for modulating gut microbiota and improving intestinal health of piglets at weaning. On the other hand, a complete revision on the nutritional profile, health benefits and application of selected fruit pomaces (apple among them) as functional foods and feeds has also been recently published (10), including clinical and preclinical studies. In general, adding fruit pomaces to foods reduces the glycemic index, increases the fiber content and total polyphenolic contents, and reduces the cooking loss, while incorporating fruit pomaces in animal feeds improves the antioxidant enzyme activities, humoral immune system, and growth performance and reduces methane emission. Pre-clinical studies with apple pomace showed a positive influence of the extract on fat loss, lipid metabolism and body weight in obese rats being fed on a high-fat diet (11). Clinical studies involved the addition of apple pomace to pure apple juice and showed a decrease in the time to arrive at maximal insulin and glucose concentration (12). Studies involving apple pomace extracts as animal feed showed a dual advantage for the livestock industry, including their use as antibiotic substitutes and conventional feedstuff alternatives. The study by Gadulrab et al. (13) on the impact of apple pomace fed dairy cattle on fatty acid concentration, ruminal fermentation, methane production, and microbial populations showed a positive effect on the ruminal metabolism such as decrease in methane emissions and ammonia concentration, along with increase in milk production, higher nutrient digestibility, and total volatile fatty acids (VFAs) in ruminant cattle.

The use of apple pomace as biostimulant for improving environmental health has also been studied by Donno et al. (14). An agro-industrial waste extract containing apple pomace was employed to determine its effects of kiwi fruit quality. Extracts showed a positive effect on the increase of fruit weight and on ascorbic acid content. Finally, apple pomace has also been tested for insect rearing as an example of circular bioeconomy (15). Through LCA studies, authors measured the potential benefits of using apple pomace to feed black soldier fly larvae. Results suggested that there are many problems to overcome before this solution is viable, mainly associated with the annual generation of wastes and its seasonality. It also shows the need for studying the regional situation of the different industries, market and region-specific integration among them.

Also interesting is the application of the residues of the insects (like for instance black soldier fly larvae) as a potential fertilizers (zoocompost). Studies demonstrate that all obtained zoocompost (employing insect feed mixtures based on potatoes, apples, corn, including sunflower meal, wheat bran or pine sawdust) significantly suppresses the development of the nematodes Meloidogyne incognita, one of the most pathogenic types of root-knot nematodes on tomatoes (16).

### 2.2 On the future One-Health implementation via Foodomics

Future applications of Foodomics to implement One-Health, e.g., addressing in-depth some of the planetary challenges described above in Figure 1, will include:

- Metabolomics for the fast and sensitive identification of the inappropriate use of antimicrobials, pesticides and insecticides.
- Foodomics in microbiome research.
- Foodomics for discovering the composition of increasingly complex products.
- €z-Foodomics as a tool for improving biosecurity.
- Foodomics for improving genetic diversity.
- Foodomics to investigate zoonotic infections (including, e.g., pathogenic pathways, transmission dynamics, diagnostic biomarkers and novel vaccines in prion, viral, bacterial, protozoan, and metazoan zoonotic infections).
- Foodomics to investigate antimicrobial resistance, the mechanisms involved and the discovery of novel treatments for antibiotic resistance.
- Foodomics in food safety (including, e.g., the detection of allergens, exposure of adulteration, identification of pathogens, and toxins).
- Foodomics combined with the transformative potential of AI in achieving the goals of One-Health.

Of course, some applications may combine several strategies, for instance, the combined use of synthetic biology technology and Foodomics to create and monitor cell factories suitable for food industry production and renewable raw material conversion into important food components, functional food additives, and nutritional chemicals. This new model of sustainable food manufacturing, based on synthetic biotechnology, will greatly: reduce the demand for (farm animal) resources and energy in food production, reduce greenhouse gas emissions, improve food production and manufacturing control, and effectively address food safety and health risks, bringing about a closer approach to One-Health goals (17). Some new applications of synthetic biology in the food industry, include alternatives to: traditional (artificial pigments, meat, starch, and milk), functional (sweeteners, sugar substitutes, nutrients, flavoring agents), and green (green fiber, degradable packing materials, green packaging materials and food traceability) foods, involving food safety, food nutrition, public health, and health-related fields. Foodomics can play a crucial role here identifying any unexpected product, confirming new foods safety or helping to understand new synthetic routes. Furthermore, with the advent of synthetic biology techniques, an increasing number of foods and ingredients will be synthesized by engineering cellular metabolic pathways. In this area, superior-quality and cost-effective production of food ingredients and nutrition factors are crucial aspects to be improved as well as the public perception of microbial foods, together with the legal, ethical, and biosecurity considerations. As mentioned, Foodomics can be a good help to better understand and overcome all these limitations.

## 3 Conclusion

As global environmental pollution increases, climate change worsens, and population growth continues, the challenges of securing a safe, nutritious, and sustainable food supply have become enormous. This has led to new requirements and trends for future food supply methods and functions. The global application of One-Health model can ensure the future food supply reducing the effects of human activity on environment and planetary boundaries, enhancing the impact on the health and well-being of humans, animals and the ecosystems we co-habit. As a summary, One-Health, Food Science and Foodomics adequately combined will enhance our understanding of food systems, promoting better health outcomes and sustainable practices. By integrating Foodomics within the One-Health framework, we will be able to better understand the complex relationships between diet, health, and the environment, leading to more effective public health strategies and sustainable food systems. Still, we have a long-way to go till we will be able to implement all the future applications of Food Science and Foodomics to make One-Health strategy routinely applied. Along the way, this implementation will expose an unexplored and challenging area of research that we have tried to anticipate for the first time in this work.

## Data Availability

The original contributions presented in the study are included in the article/supplementary material, further inquiries can be directed to the corresponding author.
